# Supplementary material for: Large-Scale Functional Assessment of Genes Involved in Rare Diseases with Intellectual Disabilities Unravels Unique Developmental and Behaviour Profiles in Mouse Models
Source: Biomedicines. 2022 Dec 6;10(12):3148. doi: 10.3390/biomedicines10123148 (PMC9775489; doi:10.3390/biomedicines10123148)
Supplement: Supplementary file 1 [file biomedicines-10-03148-s001.zip › biomedicines-2017866-supplementary(correction).pdf]

Supplementary materials

Large-Scale Functional Assessment of Genes Involved in Rare Diseases with Intellectual Disabilities Unravels Unique Developmental and Behaviour Profiles in Mouse Models

Hamid Meziane [1], Marie-Christine Birling [1], Olivia Wendling [1], Sophie Leblanc [1], Aline Dubos [1,2], Mohammed Selloum [1], Guillaume Pavlovic [1], Tania Sorg [1], Vera M. Kalscheuer [3], Pierre Billuart [4,5], Frédéric Laumonnier [6,7], Jamel Chelly [2], Hans van Bockhoven [8,9,10], and Yann Herault [1,2,*]

1 Université de Strasbourg, CNRS, INSERM, CELPHEDIA, PHENOMIN, Institut Clinique de la Souris (ICS), 1 rue Laurent Fries, 67404 Illkirch, France

2 Université de Strasbourg, CNRS, INSERM, Institut de Génétique et de Biologie Moléculaire et Cellulaire, 1 rue Laurent Fries, 67404 Illkirch, France

3 Max Planck Institute for Molecular Genetics, Research Group Development and Disease, Ihnestr. 63-73, 14195 Berlin, Germany

4 Institute of Psychiatry and Neuroscience of Paris (IPNP), Université de Paris, INSERM U1266, "Genetic and Development of cerebral cortex", 75014 Paris, France

5 GHU Paris Psychiatrie et Neurosciences, Hôpital Sainte Anne, F-75014 Paris, France

6 UMR1253, iBrain, University of Tours, Inserm, Tours, 37032, France

7 Service de Génétique, Centre Hospitalier Régional Universitaire, Tours, 37044, France

8 Department of Cognitive Neuroscience, Radboudumc, 6500 HB Nijmegen, the Netherlands.

9 Department of Human Genetics, Radboudumc, 6500 HB Nijmegen, the Netherlands.

10 Donders Institute for Brain, Cognition, and Behaviour, Centre for Neuroscience, 6525 AJ Nijmegen, the Netherlands.1

* Correspondence: herault@igbmc.fr; Tel.: +33 388 65 5715

1. Supplementary materials and methods

1.1 Embryonic phenotyping procedures

1.1.1 Viability, histology, whole mount immunostaining, alizarin red and alcian blue staining

Viability tests were performed at embryonic day 18.5 (E18.5) to monitor their capacity to survive at birth. The fetuses were weighed, placed on a warm plate at 37°C and rolled gently to stimulate them to breathe. At 30 min after extraction, the numbers of breathing animals versus cyanotic and lethargic animals were counted. Serial histological sections, whole-mount immunostaining with anti-neurofilament marker and alizarin red and alcian blue staining were performed according to standard techniques. Stained sections were digitalized using a slide scanner (Nanozoomer 2.0-HT, Hamamatsu, Japan).

1.1.2 Optical projection Tomography

Images of E10 embryos stained with anti-neurofilament marker were acquired using a custom Optical Projection Tomography system that was built according to specifications of Wong et al [53]. The tomography reconstructions were performed with the Skyscan Nrecon software, and the 3D reconstructions of the embryos were done with Avizo 9.4.0 software (Thermofisher Scientific, France)

Microcomputed tomography

The E18.5 fetuses were fixed by immersion in 10 % Formalin for a minimum of 24 hours. They were washed in 1X PBS for 24 hours (2 changes) and incubated in pure Lugol's iodine contrast agent (Sigma Aldrich ref: L6146) for 72 hours (with one change every day). Before acquisition, the samples were rinsed in 1 X PBS, quickly dried using absorbent paper and placed on the sample holder using the same orientation for each individual. Images were captured using the µCT imaging system (PerkinElmer Quantum FX, France) with a resolution of 10µm (Field of View 20) according to user's manual. Three-dimensional reconstructions were obtained using the 3D Viewer of the Quantum FX µCT software.

HREM (block en face) technology

The E15.5 fetuses were fixed in Bouin's fixative for 24 hours then washed and stored in 70 % EtOH. Fixed embryos were gradually dehydrated in an increasing series of ethanol concentrations and were embedded in a methacrylate resin (JB-4 kit, Polysciences, Warrington, PA) containing eosin and acridin orange as contrast agents as previously described [54] (see also https://dmdd.org.uk/hrem/). The resin blocks were sectioned on a HREM prototype to generate data by repeated removal of 7 µm sections. Resulting HREM data had a voxel size of 8 X 8 X 7 μm^3 and consisted of approximately 1000 aligned single images of the E15.5 whole embryos. All HREM images were converted into a volume dataset and segmented using Avizo 9.4.0 software (Thermofisher Scientific, France) to create two-dimensional and three-dimensional (3D) images. Cranial nerves, cervical vertebras and dorsal root ganglia were manually outlined using the label field function of Avizo to produce the 3D reconstructions.

1.2 Behavioural phenotyping procedures

Behavioural testing was performed in 10 to 13-week-old adults and was carried out in agreement with the EC directive 2010/63/UE86/609/CEE, and under the ethics committee accreditation number 2012-139. Most of behavioural procedures are thoroughly detailed a recent volume of current protocols [18].

Gross neurological examination:

General health and basic sensory motor functions were evaluated using a modified SHIRPA protocol (EMPRESS, eumorphia.org [55]). This analysis was adapted from that developed by Irwin [56] and from the SHIRPA protocol [57]. It provides an overview of physical appearance, body weight, body temperature, neurological reflexes and sensory abilities.

Rotarod test

This test measures the ability of an animal to maintain balance on a rotating rod (Bioseb, Chaville, France). Mice were given three testing trials during which the rotation speed accelerated from 4 to 40 rpm over 5 min. Trials were separated by 10-15 min interval. The average latency was used as index of motor coordination performance.

Grip test

This test measures the maximal muscle strength (g) using an isometric dynamometer connected to a grid (Bioseb). Each mouse was submitted to three consecutive trials immediately after the modified

SHIRPA procedure. Once the animal was holding the grid with its all paws it was slowly moved backwards until it released it.

Hot plate test

The mice were placed into a glass cylinder on a hot plate adjusted to 52°C (Bioseb). The latency of the first reaction (licking or flinches) was recorded, with a maximum of 30 sec.

Shock threshold test

The mouse was placed in the fear-conditioning chamber and allowed to habituate for 30 sec. Foot-shock was then manually applied for 1sec, and behavioral responses noted. Shock levels began at 0.05 mA, and increased in 0.05 mA steps with 30 sec interval between the shocks, until both flinch (any detectable response) and vocalization were induced. After this point, shocks were increased in 0.1mA steps until a jump (the mouse flinching such that the two hind paws leave the ground) was induced. A 1mA cut-off was employed in this test.

Circadian Activity and Ingestive Behaviours

Spontaneous locomotor activity and rears were measured using 24 individual boxes equipped with infra-red captors. The quantity of water and food consumed was measured during the test period using automated pellet feeder and lickometer (Imetronic, Pessac, France). Mice were tested for 35 hours in order to measure habituation to the apparatus as well as nocturnal and diurnal activities. Results are expressed per 1 h periods.

The open field test

Mice were tested in automated open fields (Panlab, Barcelona, Spain), each virtually divided into central and peripheral regions. The open fields were placed in a room homogeneously illuminated at 70 Lux. Each mouse was placed in the periphery of the open field and allowed to explore freely the apparatus for 30 min, with the experimenter out of the animal's sight. The distance traveled, the number of rears, and time spent in the central and peripheral regions were recorded over the test session. The number of entries and the percentage of time spent in center area were used as index of emotionality/anxiety.

Elevated plus maze

The apparatus used was completely automated and made of PVC (Imetronic, Pessac, France). It consisted of two open arms (30 X 5 cm) opposite each other and crossed by two enclosed arms (30 X 5 X 15 cm). The apparatus was equipped with infrared captors allowing the detection of the mouse in the enclosed arms and different areas of the open arms. The number of entries into and time spent in the open arms were used as an index of anxiety. Closed arm entries were used as measures of general motor activity. The number of rears in the closed arms, as well as ethological parameters such as stretching, attempts and head dips were also automatically scored.

Social recognition test:

This task was used to evaluate the preference of a mouse for a congener as compared to an object placed in an opposite compartment. Reduced social behaviour was observed in psychiatric disorders and mental retardation.

The apparatus is a transparent runway composed with a central starting box and 2 goal boxes delimited by a sliding grid at each extremity

of the runway. Testing was performed for two consecutive days. On the first day, fresh bedding was placed in the goal boxes. The mouse was placed in start box for 30 sec then allowed to explore freely the apparatus for 10 min in order to attenuate their emotionality and to evaluate any potential preference between the two compartments. On the second day, a C57Bl/6 congener from the same gender was placed in one goal box and an object (a dice for example) placed in the opposite one. The mouse was then placed in the start box for 30 sec then allowed to explore freely the apparatus for 10 min. The position of the congener and object boxes was counterbalanced to avoid any potential spatial preference. The duration of exploration of each goal box (when the mouse is sniffing the grid delimiting the goal box) was measured and the percentage of time the mouse took to explore the congener was used as index of social preference (recognition preference). The number of entries and the time spent in each goal arm, and vertical activity in each arm were also measured. The social recognition index (SR) was defined as (time Congener / (time Object + time Congener)) x100.

Auditory Startle Reflex Reactivity and Pre-Pulse Inhibition

Acoustic startle reactivity and pre-pulse inhibition of startle were assessed in a single session using standard startle chambers (SR-Lab Startle Response System, San Diego Instruments, USA). Ten different trial type were used: acoustic startle pulse alone (110-db), eight different prepulse trials in which either 70, 75, 85 or 90-dB stimuli were presented alone or preceded the pulse, and finally one trial (NOSTIM) in which only the background noise (65 dB) was presented to measure the baseline movement in the Plexiglas cylinder. In the startle pulse or prepulse alone trials, the startle reactivity was analyzed and in the prepulse plus startle trials the amount of PPI was measured and expressed as percentage of the basal startle response.

Object recognition task

The object recognition task was performed in automated open fields (see above). The open-fields were placed in a room homogeneously illuminated at 70 Lux at the level of each open field. The objects to be discriminated were a glass marble (2,5 cm diameter) and a plastic dice (2 cm). Animals were first habituated to the open-field for 30 min. The next day, they were submitted to a 10-minutes acquisition trial during which they were placed in the open-field in presence of an object A (marble or dice). The time the animal took to explore the object A (when the animal's snout was directed towards the object at a distance ≤ 1 cm) was manually recorded. A 10-minute retention trial was performed 3 h later. During this trial, the object A and another object B were placed in the open-field, and the times tA and tB the animal took to explore the two objects were recorded. The recognition index (RI) was defined as (tB / (tA + tB)) x100.

Y-maze spontaneous alternation

The apparatus was a Y-maze made of Plexiglas and having three identical arms (40x9x16 cm) placed at 120° from each other. Each arm had walls with specific motifs allowing distinguish it from the others.

Each mouse was placed at the end of one of the three arms, and allowed to explore freely the apparatus for 5min, with the experimenter out of the animal's sight. Alternations were operationally defined as successive entries into each of the three arms as on overlapping triplet sets (e.g., ABC, BCA, etc.). The percentage of spontaneous alternation was calculated as an index of working memory performance. Total arm entries

and the latency to exit the starting arm were also scored as indexes of ambulatory activity and emotionality in the Y-maze, respectively.

Pavlovian fear conditioning

Polymodal operant chambers (Coulbourn Instruments, Allentown, PA, USA) were used. Each chamber (18.5 x 18 x 21.5 cm) consisted of aluminum side walls and Plexiglas rear and front (the door) walls. A loudspeaker and a bright light constituted the sources of the cues during conditioning and cue-testing. The general activity of animals was recorded by an infrared cell placed at the ceiling of the chambers and was directly recorded on a PC computer using the Graphic State software (Coulbourn).

For conditioning, mice were allowed to acclimate for 4 min, then a light/tone (10 kHz) CS was presented for 20 s and co-terminated by a mild (1 s, 0.4 mA) footshock (US). Mice were returned to their home cages 2 min later.

Testing was performed 24 h following conditioning session. Testing for the context was performed in the morning. The mouse was placed back into the same chamber that was used for the conditioning and allowed to explore for 6 minutes without presentation of the light/auditory CS. Testing for the cue was performed in the afternoon (about 5 h after the context testing). The contextual environment of the chambers was changed (wall color, odor and floor texture). The mouse was placed in the new chamber and allowed to habituate for 2 minutes then presented with light/auditory cues for 2 minutes. This sequence was repeated once again. At the end of testing, animals were returned to their home cages.

1.3Extended statistical analysis

Raw data were centralized in a database and statistical analysis was performed using Statview and the R environment. (http://www.r-project.org/).

p-value adjustment.

Fifty-seven (57) parameters from the different behavioural tests were retained and contributed to established 10 categories of function (3-12 parameters per function, Table S2). Multiple comparisons were performed to assess a phenotype score for each biological function. To control the false discovery rate within each function, we adjusted p-value using Benjamini-Hochberg method.

Heatmap and clustering

The Heatmap.2 {gplots} R function was used to draw the heatmap and show mutant lines hierarchical clusters. Data were scaled. The distances matrix was generated using the Manhattan distance method. Agglomerative clustering was done using the complete linkage method to find similar clusters. Colors are proportional to phenotype scores.

Principal Component Analysis

The PCA {FactoMineR} R function was used to perform Principal component Analysis to explain relation between variables (biological functions) and individuals (mutant lines). Data were scaled and the interpretation of axes was based on biological functions and mutant lines:

(a) with a good quality of representation on factor map, measured by cos2. The closer a variable to the circle of correlations is, the better its representation on the factor map.

(b) with axes (or components) contribution larger than an expected average contribution.

Correlated variables were grouped together on a circle of correlations or opposite (negative correlation). The smaller an individual coordinate on the axis, the smaller its contribution to the component. The three first components explain 70.27 % of variance (Figure. 5A).

2. Supplementary Figures

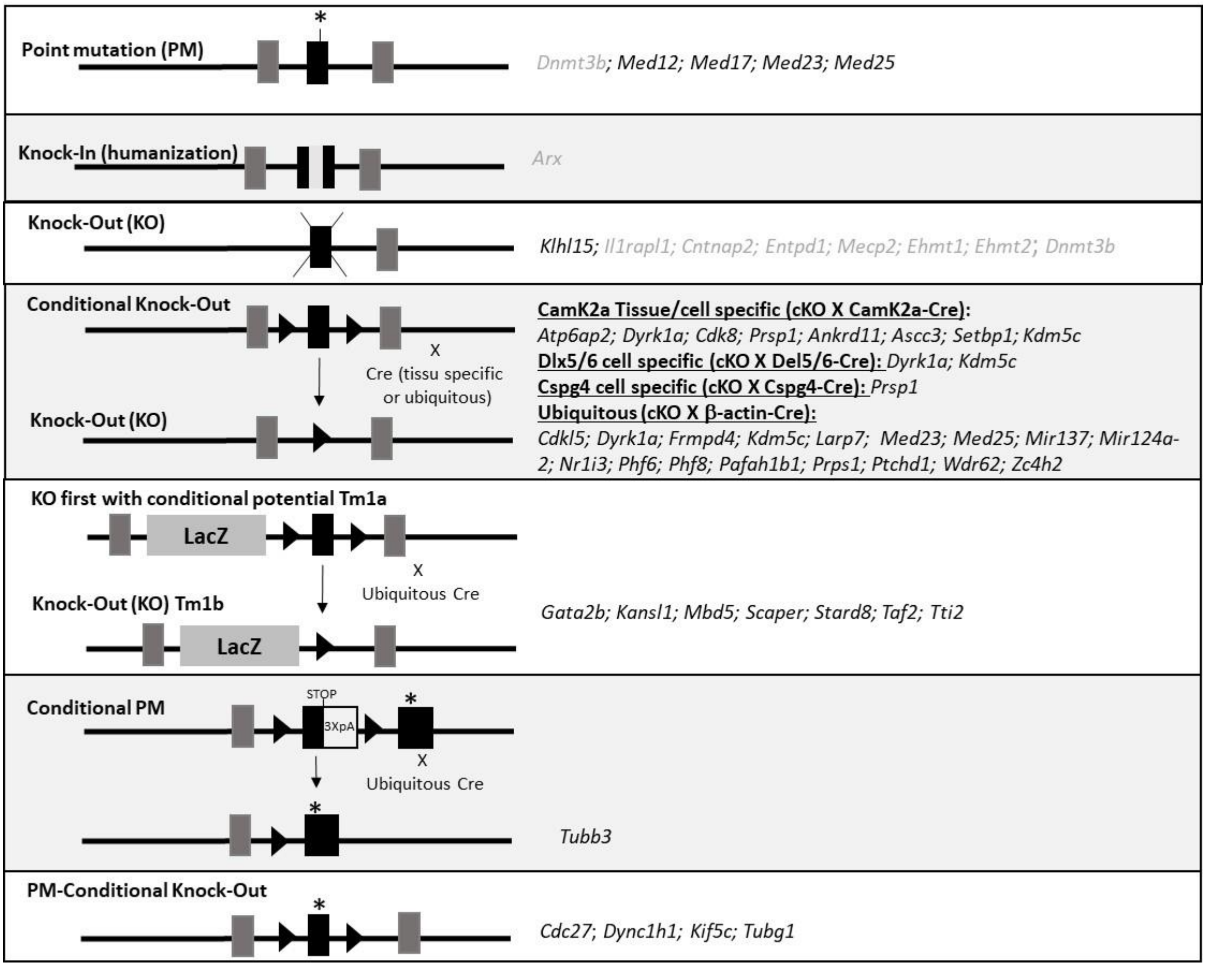

Figure S1. Mouse models generated and phenotyped in the Gencodys consortium. All ICS lines were generated (in black) in a C57BL/6N genetic background. Lines in grey are repatriated or generated at ICS in another genetic background and backcrossed in C57Bl/6N background.

Point mutations (PM) were made for *Med12, Med17, Med23, Dnmt3b, Ehmt1, Ehmt2*. Humanization of *Arx:* in light grey the humanized sequence introduced in exon 2. Complete or cell specific KO model were generated by different approaches: classical knock-out strategy by removal of one or more critical exons (*Ilr1rapl1; Cntnap2; Entpd1; Mecp2*). When available, ES cells from the IMPC consortium were used to generate the Tm1a allele. The Tm1b allele was generated by breeding the initial (Tm1a) allele with a Cre deleter line to obtain KO first alleles. If no homozygous was obtained, the conditional KO allele was generated by breeding the Tm1a allele with a Flp deleter line. A last breeding step with a cell/tissue specific Cre line was then undertaken. Conditional knock-outs (cKO) were generated in house. In the first instance, the KO allele was generated by Cre deleter breeding. If no homozygote KO could be

obtained, the mice were bred with a cell/tissue-specific Cre line (as IMPC lines). Conditional point mutation: this strategy allows the wild type allele to be expressed in a first instance. After Cre-mediated excision, the allele with the point mutation A>G leading to the mutation of a methionine into an alanine corresponding to c.1162A>G, pM388V would be expressed.

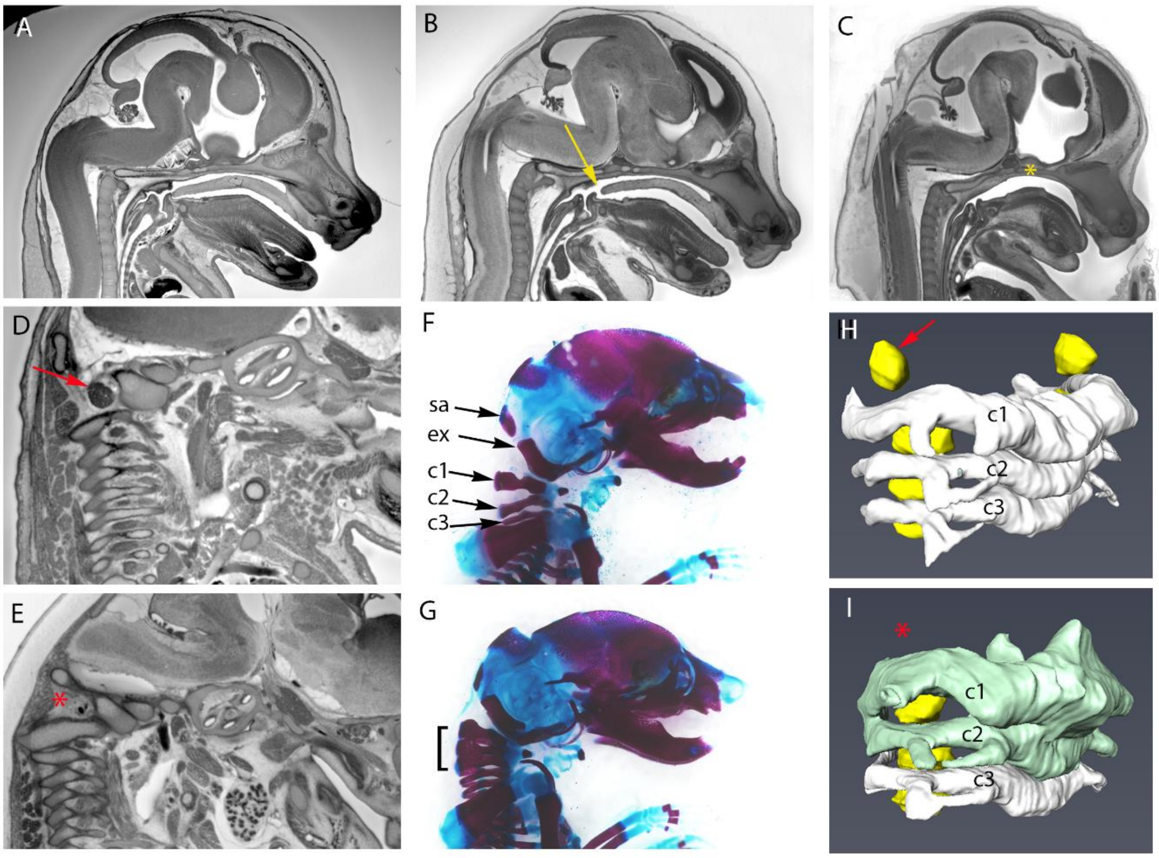

Figure S2. Cranial and cervical vertebra abnormalities of *Setbp1*$^{-/-}$ mice at E15.5 and E18.5. (A-E) High Resolution Episcopic Microscopy data through wildtype (A, D) and homozygous (B, C, E) mutant embryos at E15.5. The yellow arrow in B indicates a shorter palate with a larger nasopharyngeal opening. The yellow asterisk in B indicates a cleft palate. The red asterisk in F indicates the absence of the first pair of dorsal root. (F, G) Alizarin red (bone) and alcian blue (cartilage) staining of wildtype (F) and homozygous mutant (G) of head and cervical region at E18.5. sa: supraoccipital bone; ex: exoccipital bone; c1: 1st cervical vertebra (atlas); c2: 2nd cervical vertebra (axis); c3: 3rd cervical vertebra; the bracket in G indicate the fusion of c1, c2, c3. Segmentation and 3D reconstruction of cervical vertebras and dorsal root ganglia (yellow) of wildtype (H) and mutant (I) embryos at E15.5; fusion of c1-c2 and absence of the first pair of dorsal root (red asterisk).

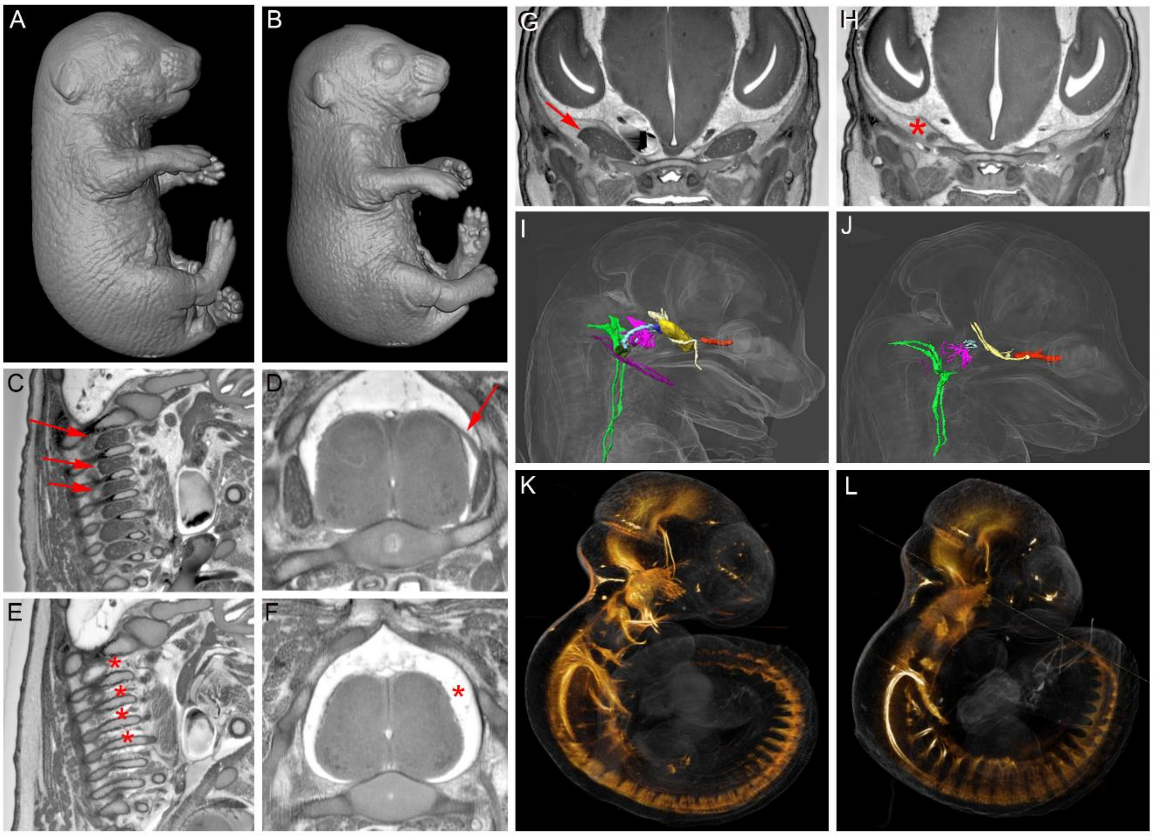

Figure S3. Cranial nerve and associated ganglia, as well as dorsal root ganglia abnormalities in *Tubb3*$^{M388V/M388V}$ mutant embryos. (A, B) Microcomputed tomography from wildtype (A) and mutant (B) fetuses at E18.5. (C-H) High Resolution Episcopic Microscopy 2D data through spinal cord and head of wildtype (C,D, G) and mutant (E, F, H) fetuses at E15.5. Asterisks in E and F indicate agenesis of the DRG and sensitive nerves, and in H agenesis of trigeminal ganglion in mutants. (I, J) Segmentation and 3D reconstruction of cranial nerves and associated ganglia of wildtype (I) and mutant (J) embryos at E15.5 (red: optic nerve (II), yellow; trigeminal ganglion (V), beige: trigeminal nerves (V), dark blue: facial ganglion (VII), light blue: facial nerve, pink: facioacoustic ganglion (VIII), green: glossopharyngeal and vagus ganglion and nerves (IX, X), purple: hypoglossal nerve (XII). (K, L) Optical projection tomography of wildtype (K) and mutant (L) embryos stained with anti-neurofilament marker at E10.5.

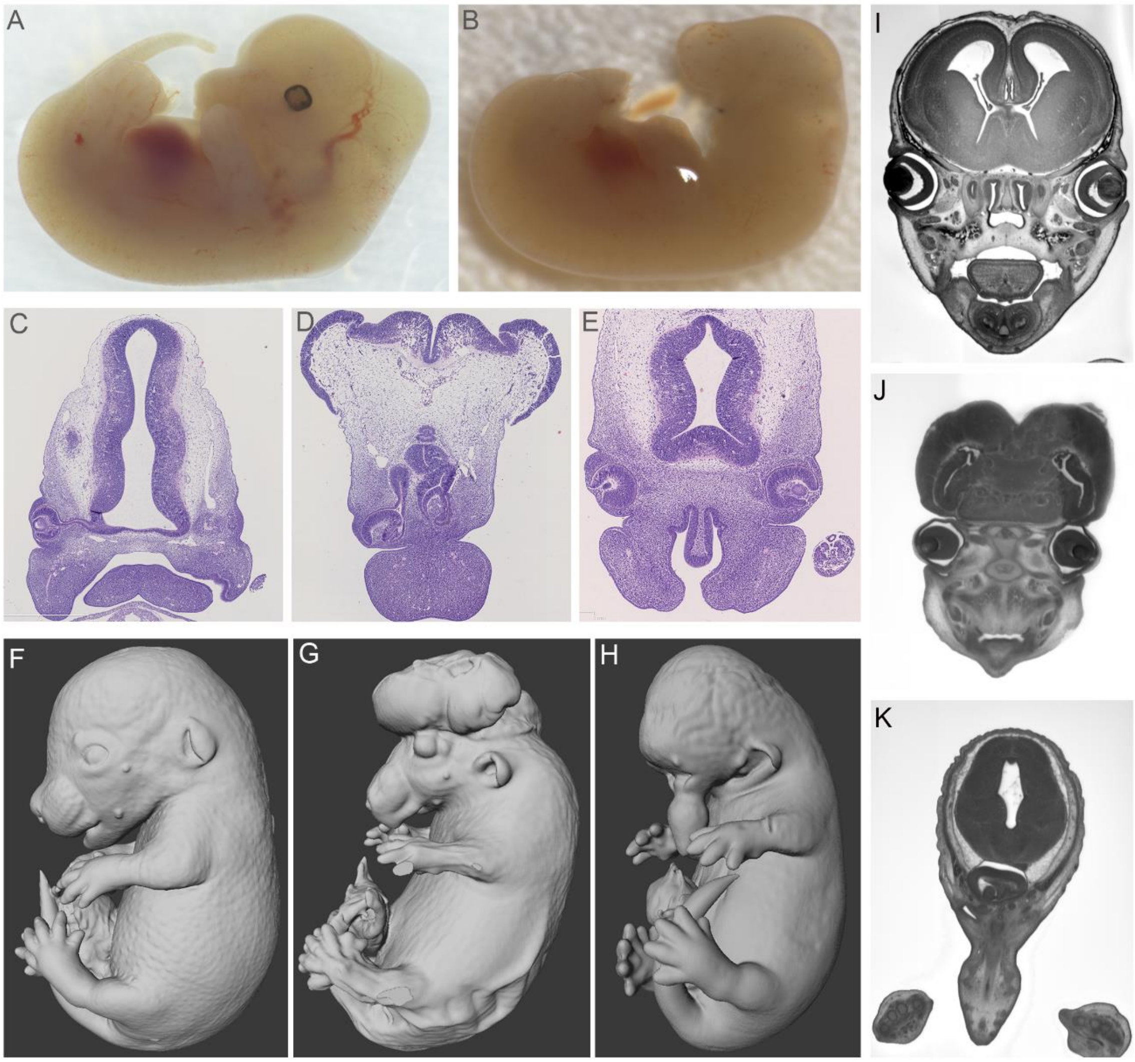

Figure S4. Severe craniofacial abnormalities in *Med25*$^{-/-}$ mutant embryos and fetuses. (A, B) External view of wildtype (A) and mutant (B) at E12.5. (C-E) Histological frontal sections through the heads of wildtype (C) and two mutant embryos (D, E) at E12.5. (F-K) High Resolution Episcopic Microscopy 3D (F-H) and 2D (I-K) data through wildtype (F, I) and homozygous (G, J; H, K) mutant embryos at E15.5.

3. Supplementary tables

Increased	Decreased	No phenotype	Not determined

	ACTIVITY												REPETITIVE BEHAVIOR								ANXIETY-R-BEHAVIOR						
Mutant Lines	CA/LA.T	CA/LA.H	CA/LA.L	CA/LA.N	OF/LA	Shirpa/LA	Ymaze/LA	EPM/LA	Soc. Recog/LA1	Soc. Recog/LA2	Fear/LA H1	Fear/LA H2	CA/Rears.T	CA/Rears.H	CA/Rears.L	CA/Rears.N	OF/ Rears 5	OF/ Rears T	Soc. Recog/rears 1	Soc. Recog/rears 2	OF/ Nentries	OF/ % time 5 min	OF/ % time T	EPM/HD	EPM/%entries	EPM/% time	YMaze /lat
Cdk8 ^{Camk2a}	0.88	0.15	0.94	0.36	<0.002	0.07	<0,05	0.37	0.51	0.26	0.47	<0.05	0.16	0.08	0.98	0.23	0.22	0.09	0.74	0.12	<0.01	<0.001	0.14	0.43	0.47	0.18	0.52
Ankrd11 ^{Camk2a}	0.37	0,8	0,53	0,18	<0.001	0.62	<0.05	<0.05	0.24	0.38	<0.05	<0.05	0.14	0.16	0.47	0.16	<0,05	<0.05	0.71	0.52	<0.01	<0.05	0.08	0.06	0.13	0.18	0.29
Atp6ap2 $^{Camk2a/y}$	<0.01	<0.01	0.31	<0.05	<0.001	0.47	<0.01		0.07		0.06	0.15	<0.01	<0.01	0.20	0.052	0.16	<0.001	0.78		0.001	0.82	0.32				0.50
Il1rapl1 $^{-/y}$	<0.01	<0.01	0.17	<0.01	<0.01	<0.01	<0.001				1	0.20	<0.001	<0.001	0.14	<0.01	0.07	0.51			0.28	<0.001	0.71				0.18
Prps1 $^{Camk2a/y}$	0.75	0.55	0.49	0.90	<0.05	0.56	0.17	0.70	0.15	0.18	0.77	0.25	0.29	0.17	0.82	0.78	0.92	0.07	0.47	0.32	<0.01	0.92	<0.01	0.43	0.58	0.89	0.32
Ptchd1 $^{-/y}$	<0.001	<0.001	<0.001	<0.001	<0.05	0.65	0.39	0.48	p<0.001	p<0.001	0.12	<0.01	<0.05	<0.05	0,1	<0.05	<0.05	<0.01	0.37	0.13	<0.01	<0.01	<0.05	0.55	0.10	<0.05	0.055
Arx $^{Dup24/y}$	0.47	<0.05	0.23	0.86	p<0.05	0.15	<0.01	0.82	0.10		0.13	0.052	0.06	0.09	0.11	0.10	0.08	<0.01	0.25		0.07	<0.01	0.27	0.27	0.58	0.56	0.47
Ascc3 ^{Camk2a}	0.05	0.11	0.29	<0.05	0.16	<0.05	0.49	<0.001	0.28	0.54	0.83	0.40	0.12	0.15	0.82	0.08	0.13	0.25	<0.05	0.06	0.76	0.19	0.35	<0.01	<0.07	0.07	0.08
Prps1 $^{Cspg4/y}$	0.08	0.11	0.16	0.56	0.06	0.10	0.53	0.09	0.55	0.69	0.37	0.53	0.30	0.24	0.30	0.76	0.32	0.30	0.80	0.94	0.23	0.47	0.52	0.29	0.52	0.22	0.27
Nr1i3 $^{-/-}$	0.72	0.74	0.95	0.77	0.65	0.07	0.29	0.36	0.78	0.96	0.97	0.46	0.43	0.30	0.44	0.85	0.27	0.61	0.38	0.34	0.82	0.85	0.13	0.32	0.81	0.90	0.89
Dyrk1a $^{Dlx5-6/+}$	0.88	0.87	0.41	0.86	0.25	<0.05	0.08	0.11	0.35	0.36	0.18	0.10	0.40	0.50	0.22	0.38	0.19	0.26	0.89	0.95	0.97	0.65	0.73	0.41	0.51	0.30	0.23
Dyrk1a ^{Camk2a}	0.35	0.40	0.70	0.29	0.23	0.66	0.68	0.58	0.26	0.77	0.86	0.56	0.45	0.42	0.69	0.45	0.30	0.96	0.17	0.28	0.06	0.45	<0.04	0.31	0.14	0.13	0.09
Stard8 $^{-/y}$	0.49	0.82	0.38	0.37	0.72	0.98	0.27	0.34	0.89	0.94	1	0.59	0.17	0.12	0.69	0.31	0.24	0.89	0.36	0.73	0.36	0.35	0.20	0.27	0.97	0.33	<0.05
Med17 $^{L369P/+}$	0.41	0.39	0.16	0.87	0.15	0.60	0.40	0.98	0.69	0.32	0.26	0.49	0.57	0.74	0.98	0.32	0.051	<0.01	0.96	0.25	0.06	0.07	0.08	0.97	0.21	0.90	0.90
Wdr62 $^{-/-}$	0.53	0.16	0.26	0.76	0.25	0.58	0.92	0.13	0.92	0.36	0.55	0.56	0.52	0.89	0.31	0.35	0.90	0.89	0.88	0.26	0.08	<0.05	0.17	0.31	0.13	0.13	0.47
Kif5c $^{+/-}$	0.57	0.53	0.77	0.58	0.41	0.12	0.45	0.52	0.63	0.93	0.55	0.94	0.95	0.44	0.78	0.52	0.22	0.98	0.56	0.75	0.43	0.29	0.55	0.88	0.64	0.84	0.01
mIR137 $^{+/-}$	0.33	0.50	0.66	0.21	0.42	0.48	0.13	0.55	0.45	0.62	0.68	0.46	0.13	0.12	0.47	0.20	0.06	0.30	0.43	0.24	0.60	0.21	0.17	0.01	0.06	<0.05	0.21
Entpd1 $^{-/-}$	0.39	0.56	0.06	0.44	0.44	<0.01	<0.05	<0.04	<0.05	<0.01	0.12	0.20	0.98	0.44	0.36	0.88	0.52	0.26	0.07	<0.05	0.89	0.32	0.65	0.12	0.47	0.76	0.92
Ehmt2 $^{+/-}$	<0.01	0.10	0.06	<0.01	0.63	0.15	0.20	0.21	0.45	0.73	0.47	0.49	0.31	0.71	0.09	0.23	0.08	0.68	0.24	0.49	0.72	0.33	0.76	0.052	0.99	0.52	0.18
Cntnap2 $^{-/-}$	0.83	0.74	0.28	0.62	<0.05	0.01	0.11	<0.001	<0.01	0.10	0.50	0.52	0.051	0.21	0.29	<0.05	0.052	<0.05	0.24	0.30	<0.05	01;056	0.22	0.51	0.43	0.22	0.55
Dyrk1a $^{+/-}$	0.89	0.63	0.59	0.76	<0.05	<0.01	0.68	0.63	0.83	0.57	0.52	0.42	0.90	0.73	0.60	0.49	0.41	<0.05	0.84	0.53	0.28	0.70	0.32	0.11	0.25	0.25	0.42
Setbp1 $^{+/-}$	0.29	0.08	0.06	0.64	0.97	0.39	0.79	0.98	0.25	0.99	<0.05	<0.05	0.76	0.13	0.08	0.24	0.14	<0.05	0.08	0.67	0.40	0.91	0.121	0.43	0.43	0.33	0.10
Mecp2 $^{-/y}$	<0.05	0.98	<0.01	<0.01	0.67	0.19	0.24	<0.01	0.15	0.45	0.30	0.94	<0.05	0.28	<0.05	<0.05	0.23	0.18	0.48	0.98	0.62	0.52	0.21	<0.05	0.09	0.06	0.40
Mbd5 $^{+/-}$	<0.05	<0.01	0.35	0.37	<0.05	0.42	0.051	<0.001	<0.001	<0.01	0.12	0.56	0.07	1.00	0.83	<0.01	0.10	0.33	<0.01	0.19	0.01	0.13	<0.05	<0.05	0.92	0.68	<0.001
Ehmt1 $^{+/-}$	0.14	<0.05	0.84	0.61	<0.05	0.42	0.16	0.22	<0.01	<0.05	0.053	<0.01	<0.05	<0.05	0.16	0.17	<0.001	<0.01	0.11	<0.05	<0.01	<0.001	0.10	0.25	0.29	0.61	0.80
Ehmt1 $^{+/-}$/Ehmt2	0.84	0.59	0.66	0.85	<0.01	0.08	0.05	<0.05	0.25	<0.05	0.12	0.01	0.32	0.16	0.07	0.75	0.001	0.01	0.17	<0.001	<0.05	<0.05	<0.05	0.20	0.33	0.73	0.66
Cdkl5 $^{-/y}$	<0.001	0.20	0.07	<0.001	0.44	<0.001	<0.01	0.50	<0.05	0.13	<0.01	0.34	<0.05	0.57	0.17	<0.01	<0.05	0.25	<0.01	<0.05	0.30	<0.01	<0.05	<0.01	0.43	0.45	<0.001

	EXPLORATION OF NOVELTY				SENSORIMOTOR REPONSE				MOTOR ABILITIES					SOCIAL BEHAVIOR				LEARNING / MEMORY							SENSORIMOTOR GATING			PAIN SENSITIVITY		
Mutant Lines	CA/LA.21h	CA/Rears.21h	OF/LA 5min	OR/exploration	Startle 65	Startle 70	Startle 85	Startle 110	grip strength m	grip strength R	Rotarod /latency	Rotarod/latency T1	Shirpa/Tremors	Soc. Recog/preference % duration	Soc. Recog/memory % duration	Soc. Recog/preference % nb	Soc. Recog/memory % nb	Context fear cond 1	Context fear cond 2	Context fear cond 3	Cued fear cond 1	Cued fear cond 2	OR/recognition	Ymaze/working mem	PPI G	PPI 70	PPI 85	Hpt plate	Shock threshold J	Shock threshold V
Cdk8 ^{Camk2a}	0.13	<0.03	<0.01	0.73	0.87	0,54	0.08	0.05	0.11	<0.04	0.75	0.57	1	0.18	0.58	0.10	0.24	0.10	0.19	01.26	0.06	0.56	0.27/0.62	0.54	0.50	0.97	0.12	0.56	0.44	0.40
Ankrd11 ^{Camk2a}	<0.01	0.15	<0.001	0.16	0.68	0.61	0.98	0.47	0.90	0.94	0.69	0.48	1	0.38	0.18	0.85	0.93	<0.05	0.07	<0.05	0.16	0.05	0,72/<0,05	0.16	0.67	0.91	0.63	0.96	0.43	0.67
Atp6ap2 $^{Camk2a/y}$	<0.001	<0.001	<0.01	<0.01	<0.01	<0.01	0.06	<0.05	0.43	0.13	0.45	0.42	1	0.93		0.43		0.08	0.07	0.01	0.12	<0.01	0.74/<0.01	0.83	0.51	0.58	0.23	0.93	0.04	<0.05
Il1rapl1 $^{-/y}$	<0.01	<0.001	0.27	<0.01	0.39	0.72	0.44	0.34	0.18	0.08	0.27	0.40	1					<0.001	<0.01	<0.05	0.052	<0.05	0.67/<0.05	0.24	0.25	0.60	0.66	0.93	0.58	0.34
Prps1 $^{Camk2a/y}$	0.07	p<0.001	0.56	0.42	0.73	0.68	0.10	0.15	0.13	0.90	0.67	0.98	1	0.79	0.052	0.60	0.15	0.85	<0.05	0.30	0.67	0.14		<0.05	0.32	0.43	0.37	0.07	0.65	0.73
Ptchd1 $^{-/y}$	<0.001	<0.05	<0.05	0.26	0.16	0.43	0.70	0.06	0.09	0.01	0.63	0.37	1	0.97	0.12	0.56	0.88	0.11	0.22	0.16	0.49	0.18	0.37/0.14	<0.05	0.19	0.99	<0.01	0.29	0.66	0.44
Arx $^{Dup24/y}$	0.54	0.63	0.19	0.65	0.25	0.99	0.36	0.72	0.66	0.29	<0.05	0.06	1	0.37		0.86		<0.05	<0.05	<0.01	0.69	0.63	0.25/<0.01	0.08	0.36	0.53	0.47			
Ascc3 ^{Camk2a}	<0.05	0.12	<0.01	0.30	0.39	0.90	0.77	0.24	0.33	0.57	0.26	<0.05	1	0.71	0.79	0.15	0.63	0.87	0.82	0.72	0.08	0.51		0.18	0.47	0.73	0.76	0.12	0.29	<0.05
Prps1 $^{Cspg4/y}$	0.07	0.25	0.24	0.09	0.69	0.56	0.80	0.94	0.23	<0.05	<0.05	<0.05	1	0.09	0.06	0.11	0.61	0.12	0.10	0.57	0.50	0.15		0.35	0.23	0.63	0.11	0.28	0.90	0.48
Nr1i3 $^{-/-}$	0.81	0.39	0.68	0.84	<0.05	0.24	0.22	0.13	0.63	0.29	0.62	0.74	1	0.14	0.15	0.43	0.68	0.49	0.05	<0.05	0.36	0.21		0.54	0.59	0.70	0.66	0.88	0.72	0.58
Dyrk1a $^{Dlx5-6/+}$	0.54	0.45	0.08	<0.05	0.40	0.66	0.45	0.71	0.46	0.39	<0.05	0.35	1	0.79	0.26	0.78	0.87	0.70	0.50	0.42	0.58	0.27	0.79/<0.05	0.77	0.25	0.17	0.97	0.19	0.07	0.58
Dyrk1a ^{Camk2a}	0.86	0.64	0.97	0.06	0.25	0.41	0.18	0.54	<0.05	0.08	<0.05	0.40	1	0.84	0.12	0.61	<0.01	<0.05	<0.05	0.13	0.95	0.68	<0.05/<0.05	0.86	0.70	0.52	0.99	<0.05	0.70	0.89
Stard8 $^{-/y}$	0.29	<0.05	0.12	0.84	0.68	0.60	0.91	0.59	0.63	0.52	0.10	0.11	1	0.21	0.45	<0.05	0.40	0.28	0.14	0.98	0.36	0.59		0.32	0.15	0.31	0.18	0.33	0.20	0.99
Med17 $^{L369P/+}$	0.38	0.49	0.12	0.80	0.84	0.29	0.84	0.75	0.10	0.54	0.77	0.45	1	0.40	0.58	0.90	0.62	0.15	0.92	0.67	0.54	0.63		0.98	0.57	0.81	0.58	0.14	0.44	0.67
Wdr62 $^{-/-}$	0.94	0.96	0.40	0.88	0.24	0.55	0.93	0.49	0.99	0.56	0.67	0.34	1	0.81	0.13	0.69	0.47	0.30	0.50	0.70	0.95	0.60		0.72	0.45	0.64	0.30	0.41	<0.001	0.57
Kif5c $^{+/-}$	0.19	0.78	0.18	0.79	0.75	0.60	0.14	0.82	0.59	0.66	0.96	0.58	1	0.36	0.51	0.60	0.37	0.12	0.68	0.12	0.29	<0.01		0.06	0.42	0.83	0.38	0.56	0.16	0.82
mIR137 $^{+/-}$	0.40	0.42	<0.05	<0.001	0.88	0.33	0.90	0.15	0.69	0.38	0.054	0.18	1	0.81	0.20	0.66	0.64	0.21	0.59	0.90	0.73	0.69		0.13	<0.01	<0.01	0.35	<0;05	0.01	1
Entpd1 $^{-/-}$	0.90	0.97	0.97	0.24	0.23	0.07	<0.05	0.10	0.77	0.85	<0.01	<0.001	1	0.78	0.62	0.70	0.21	0.15	0.06	0.52	0.78	0.25		<0.05	0.14	0.08	0.23	0.26	0.58	0.37
Ehmt2 $^{+/-}$	0.46	0.16	<0.05	0.30	0.22	0.65	0.95	0.07	0.19	0.42	0.48	0.49	1	0.60	<0.05	0.44	0.21	0.31	0.72	0.36	0.85	0.23	0.94/0.07	0.71	<0.05	0.11	0.46	0.73	0.72	0.29
Cntnap2 $^{-/-}$	<0.05	0.37	<0.01	0.48	0.35	0.37	<0.05	0.26	0.63	0.62	0.88	0.99	1	0.80	0.52	0.78	0.64	0.07	0.36	0.23	0.50	<0.05	0.74/0.06	0.56	0.21	<0.05	0.44	<0.05	0.93	0.25
Dyrk1a $^{+/-}$	<0.05	<0.05	0.28	0.07	0.15	0.22	0.35	0.35	<0.01	0.12	0.26	0.57	1	0.92	0.08	0.59	<0.05	0.47	0.76	0.80	0.50	0.77		0.75	0.07	0.27	<0.01	<0.05	0.99	0.21
Setbp1 $^{+/-}$	0.70	0.93	0.36	0.11	0.64	0.80	0.65	<0.001	<0.01	<0.05	0.63	0.46	1	0.42	0.57	0.95	0.15	0.52	0.32	0.13	<0.05	0.08		0.71	0.17	0.90	<0.01	0.30	0.22	0.06
Mecp2 $^{-/y}$	0.48	0.28	<0.05	ND	0.82	0.37	<0.001	<0.05	<0.05	0!;34	0.35	0.10	<0.001	0.89	0.07	0.86	0.77	0.83	0.69	0.67	0.08	0.20		0.43	<0.05	0.28	<0.05	<0.001	0.83	0.88
Mbd5 $^{+/-}$	0.67	0.81	<0.05	0.79	0.91	0.77	0.21	0.001	0.18	0.69	<0.05	<0.05	1	<0.01	0.10	0.22	0.43	<0.05	0.054	0.19	0.08	<0.01		0.82	0.26	0.67	<0.05	0.06	0.24	0.47
Ehmt1 $^{+/-}$	0.29	<0.05	<0.001	<0.001	0.58	0.77	0.45	<0.01	0.051	0.49	0.44	0.54	1	0.27	0.24	0.29	0.52	0.36	0.79	0.58	0.61	0.39	<0.05/0.76	0.91	0.10	0.45	0.06	0.28	0.68	0.61
Ehmt1 $^{+/-}$/Ehmt2	0.51	<0.05	<0.001	<0.001	0.43	0.48	0.69	<0.01	0.31	0.42	0.19	0.47	1	0.85	0.18	0.64	<0.01	0.93	0.74	0.94	0.87	0.89	0.07/0.56	0.58	0.051	0.33	<0.05	0.09	0.56	0.41
Cdkl5 $^{-/y}$	<0.01	0.39	<0.05	1	0.40	0.21	0.28	0.20	<0.01	0.07	0.41	0.66	1	0.91	0.95	<0.01	0.90	0.13	0.96	<0.05	0.25	0.1		0.60	<0.01	0.16	<0.05	<0.01	1	<0.01

Table S1. Gene/phenotype heatmap drawn from main parameters of different tests from 27 characterized mutant lines. The pheno-hits are defined based on statistical differences between WTs and mutants for the majority of parameters ($p<0.05$). For object recognition performance, the two values indicate comparisons of recognition index between WT and mutant, and comparison of mutant performance to the chance level (no difference of exploration between the familiar and the novel object; absence of significance is considered as random performance and thus altered object recognition).

Biological function	Activity	Anxiety related behavior	Exploration of novelty	Learning memory	Motor abilities	Pain sensitivity	Repetitive behavior	Sensori-motor response	Sensori-motor gating	Social behavior
Parameters	CA-L.A. Hab	OF- % time in center 5 min	CA-L.A. 21H	OR-Recog-nition index	Grip strength	ST-Vocalization intensity	CA-R.A. Hab	Startle-BN (65)	%PPI-PP70	social preference (%) -sniffing duration
	CA-L.A. Night	OF- % time in center Tot	CA-R.A. 21H	FC-% cont-1	Grip strength (adjusted)	ST-Jump intensity	CA-R.A. Night	Startle-P70	%PPI-PP85	Social memory (%) - sniffing duration
	CA-L.A. Light	OF Nb entries in center	OF- Dist. 5 min	FC-% cont-2	Shirpa-Tremor	HP-latency	CA-R.A. Light	Startle-P85	%PPI-Glb	Social preference(%) - nb of sniffings
	CA-L.A. Total	YM-Latency	OR-Exploration During acquisition	FC-% cont-3	RR-Trial 1 latency		CA-R.A. Total	Startle-ST110		Social memory (%) - nb of sniffings
	Shirpa-L.A.	EPM-% open time entries		FC-% cue-1	RR-Mean latency		OF-Nb rear. 5 min			
	OF-Distance	EPM-Head-Dips		FC-% cue-2			OF-Nb rear. total			
	FC-immobility hab 1	EPM-% open time		YM-% SPA				SR-Rears 1<br>Rears - activity1		
	FC-immobility hab 2						SR-Rears 2			
	YM-Nb entries									
	EPM-Closed Arm Entries									
	SR-Nb entries 1									
	SR-Nb entries 2									

CA: circadian activity test
OF: open field test
FC: fear conditioning test
YM: Y-maze test
EPM: elevated plus maze test

SR: Social recognition test
OR: object recognition test
RR: rotarod test
ST: shock threshold
HP: hot plate

L.A. : Locomotor activity
R.A. : Rearing activity
hab : habituation
cont : context

Table S2. Biological function categories and related parameters, established for PCA analysis and cluster representation.

	Activity	Anxiety related behavior	Exploration of novelty	Learning memory	Motor abilities	Pain sensitivity	Repetitive behavior	Sensorimotor response	Sensorimotor gating	Social behavior
Il1rapl1$^{-/y}$	0.50	0.14	0.75	0.29	0	0	0.38	0	0	0
ARX$^{Dup24/y}$	0.08	0	0	0.14	0	0	0.13	0	0	0
Mecp2$^{-/y}$	0.25	0	0	0	0.20	0.33	0	0.25	0.67	0
Atp6ap2$^{CamK2a/y}$	0.42	0.14	1	0.29	0	0.33	0.38	0.75	0	0
Cntnap2$^{-/-}$	0.25	0	0.25	0	0	0	0	0	0	0
Nr1i3$^{-/-}$	0	0	0	0	0	0	0	0	0	0
Cdkl5$^{-/y}$	0.42	0.43	0.50	0	0.20	0.67	0.50	0	0.67	0
Ptchd1$^{-/y}$	0.67	0.29	0.50	0	0.20	0	0.13	0	0.33	0
Wdr62$^{-/-}$	0	0	0	0	0	0.33	0	0	0	0
Dyrk1a$^{CamK2a/Camk2a}$	0	0	0	0	0	0	0	0	0	0
Dyrk1a$^{Dlx5\text{-}6/+}$	0	0	0	0	0	0.67	0	0	0	0
Entpd1	0.25	0	0	0	0.40	0	0.13	0	0	0
Cdk8$^{CamK2a/Camk2a}$	0.08	0.29	0.50	0	0	0	0	0	0	0
Prps1$^{CamK2a/y}$	0	0.29	0.25	0	0	0	0	0	0	0
Prps1$^{Cspg4/y}$	0	0	0	0	0	0	0	0	0	0
Setbp1$^{-/+}$	0	0	0	0	0.40	0	0	0.25	0.33	0
Ankrd11$^{CamK2a/Camk2a}$	0.08	0.14	0.50	0	0	0	0	0	0	0
EHMT1$^{-/+}$	0.17	0.29	0.75	0	0	0	0.38	0.25	0	0
mIR137$^{-/+}$	0	0	0.25	0	0	0.33	0	0	0.33	0
Kif5c$^{-/+}$	0	0	0	0	0	0	0	0	0	0
Mbd5$^{-/+}$	0.33	0.43	0	0.14	0.40	0	0.13	0.25	0	0.25

Table S3. The phenotype scores calculated for each mutant line and functional category to perform PCA analysis and cluster representation.
